# A comparative study on teaching satisfaction of smart class and traditional class in pharmacy administration

**DOI:** 10.1186/s12909-023-04676-5

**Published:** 2023-09-26

**Authors:** Qingyu Wang, Yan Gao, Juan Du

**Affiliations:** 1 Department of Pharmacy Administration, College of Pharmaceutical Sciences, Zhengzhou University, Zhengzhou, 450001 China; 2 Department of Pharmacy Administration, Institute of Drug Discovery and Development, Zhengzhou University, Kexue Road 100, Zhengzhou, 450001 Henan Province China

**Keywords:** Smart class, Traditional class, Satisfaction, Comparative study

## Abstract

**Objective:**

To compare the difference in teaching satisfaction between traditional classes and smart classes after adopting the smart class design for “Pharmacy Administration”.

**Methods:**

20 classes were selected for traditional class teaching and smart class teaching, respectively. The first 10 classes were implemented using traditional teaching methods, and the last 10 classes were implemented using smart classes. After each 10 classes, the ten-point Likert scale was used to measure teacher satisfaction and course satisfaction, and the Mann-Whitney U test was performed on the mean value of satisfaction.

**Results:**

The mean and standard deviation of teacher satisfaction using traditional classes (n = 193) were 9.82 ± 0.471, and the mean and standard deviation of teacher satisfaction using smart classes (n = 199) were 9.85 ± 0.566, P > 0.05; the mean and standard deviation of course satisfaction using traditional classes (n = 193) were 9.68 ± 0.636, and the mean and standard deviation of course satisfaction using smart classes (n = 199) were 9.75 ± 0.649, P > 0.05.

**Conclusion:**

After using the smart class teaching practice, the scores of teacher satisfaction and course satisfaction improved; the mean of teacher satisfaction increased by 0.03 points and the mean of course satisfaction increased by 0.07 points. For course satisfaction and teacher satisfaction, there is no significant difference between using traditional class and smart class.

## Background

“Pharmacy Administration” is one of the compulsory courses for undergraduate pharmaceutical education in colleges and universities, and one of the subjects for professional qualification examinations for Licensed Pharmacists and Licensed Traditional Chinese Pharmacist. This course is closely related to the development of pharmaceutical occupations in the future. It is the necessary professional knowledge and basic laws and regulations that pharmacist should acquire engaged in the pharmaceutical industry. In recent years, with the New “Chinese Medicine Law of the People’s Republic of China” (revised 2017), the “Pharmaceutical Administration Law of the People’s Republic (2019 revised), the “Vaccine Administration Law of the People’s Republic of China” (2019 revised), the “Drug Registration Regulation) “(Amendment of 2020), the implementation of “the Regulations for the Supervision and Management of Drug Production and Supervision ”(Amendment of 2020), and most of the content of the textbook has been modified. On the other hand, the content of the subjects of the " Pharmacy Administration and Regulations” of the Licensed Pharmacist Exam also changed significantly, the exam form is more flexible and diverse. And comprehensive knowledge analysis tests become more and more difficult, which are adapted from real cases in the practice of drug supervision and management and examine the students’ ability to analyze and solve practical problems using their knowledge of pharmaceutical laws and regulations. Therefore, it is necessary and urgent for teachers to change the teaching methods of the Pharmacy Administration.

The way students acquire knowledge and skills is gradually changing as mobile internet and computer technology advance. With the spread of COVID-19 (Coronavirus Disease 2019), online teaching has emerged as a new method to supplement the commonly used offline teaching, and the smart classroom teaching mode has emerged. According to Lu YF [1] (2020), a smart class is one that promotes personality learning, cooperation and mutual assistance, and intelligent analysis, thereby effectively promoting the improvement of students’ knowledge and skills through the creation of a smart learning environment. Zhao QQ et al. [2] (2021) developed a web-based information technology-based smart classroom teaching model for basic medical chemistry. Students were more satisfied, with the average comprehensive assessment score of students who participated in the smart classroom teaching reform being 3.4 points higher than students in other parallel classes such as dentistry and public health. Yun Q et al. (2020) [3] used a randomized controlled trial method for a junior medical student radiology course, with the experimental group using smart-class (the smart-class group) and the control group using the traditional teaching method, and found that the Smart-Class group had higher mean quiz scores (r = 0.4, p = 0.001) and final exam application scores (r = 0.3, p = 0.005) than the traditional group. Liu FY et al. (2019) [4] analyzed and summarized 123 articles of smart classroom literature published in core journals from 2010 to 2018 on The China Knowledge Network Database, with 119 articles of smart class in social science, 18 articles in information technology, and 4 articles in philosophy and humanities, respectively, and a weak combination of smart class in medicine. Liu, Feng-Yuan think that promoting deep integration between smart class and medical education can help students improve their innovative thinking ability. Zhang L, Zhu SL (2021) [5] conducted a review of the domestic Smart Class research literature (2015–2019). The extensive research and application of smart classrooms is an unavoidable trend. The smart class is characterized by new objectives, new processes, and new assessments. Future research topics that need to be broken through include the systematic design and practical demonstration of smart class teaching models, strategies for generating student creativity in smart classrooms, and evaluating the effectiveness of developing student competencies.

The reform of pharmacy management course teaching based on interaction between teachers and students in the smart class is in line with the future educational trend. It has more advantages than the traditional teaching model, but research on satisfaction and consequence evaluation is still lacking. It has become very important to investigate and explore smart class teaching methods in practice in order to adapt to the future pharmacy professional education. In the pharmacy management course at the College of Pharmaceutical Sciences, Zhengzhou University, a longitudinal study on the satisfaction evaluation between the smart class and the traditional class was conducted.

## Methods

### Research design and reform content

**Fig. 1:**
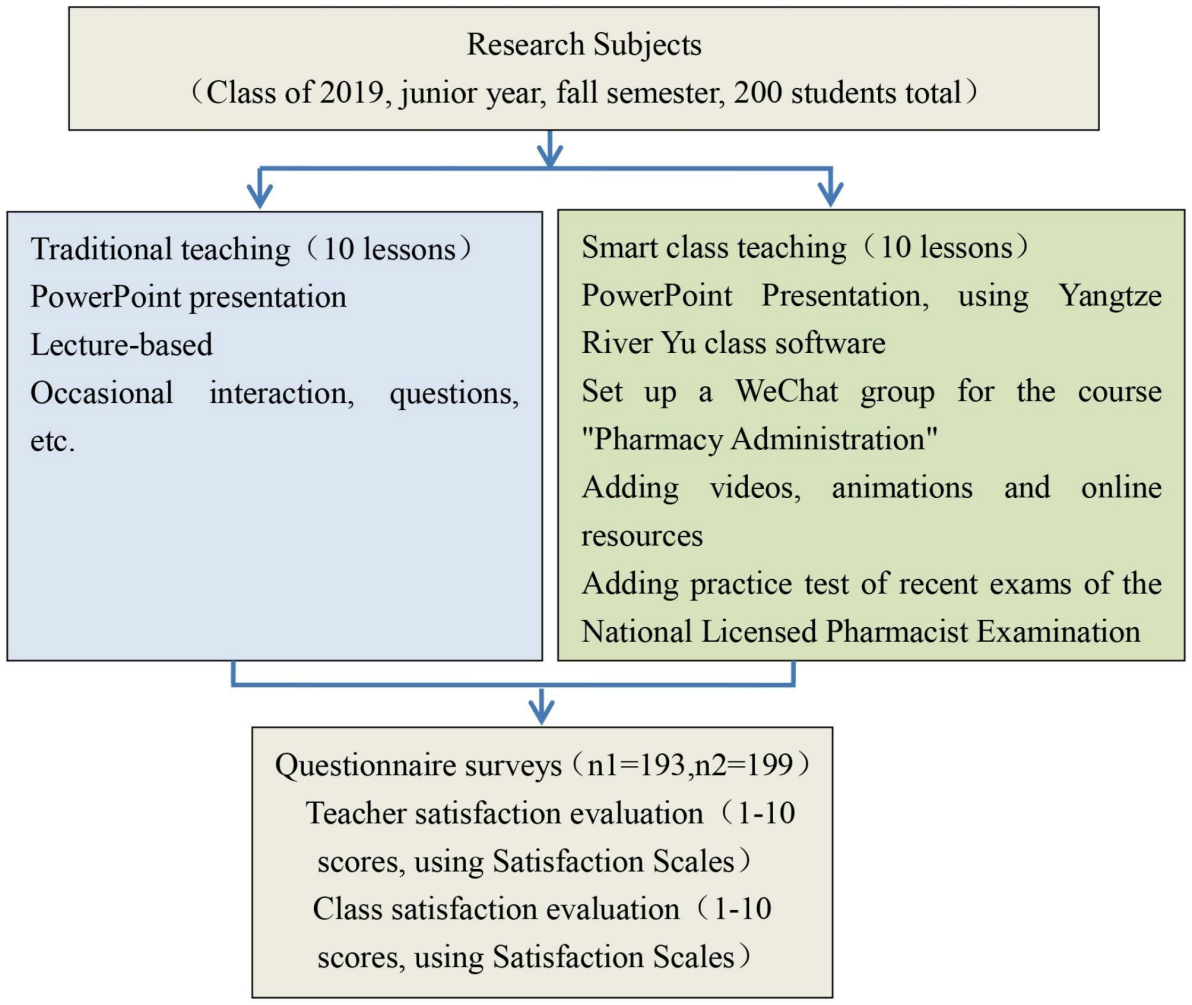
Diagram of teaching reform research

20 lessons were chosen to evaluate the effectiveness of traditional class teaching and smart class teaching. Traditional teaching techniques are used in the first 10 classes, while smart class reform is implemented in the final 10 lessons. Utilizing the Yangtze River Yu class software, opening the Course WeChat group to send learning materials, responding to questions, etc., increasing class interaction, increasing the question of case analysis, adding exercises from prior National Licensed Pharmacist Examination for each lesson, utilizing the Online School resources supported by the Yangtze River Yu class, and adding relevant chapters are the main components of smart class reforms. A satisfaction survey is carried out after the end of each session. See Fig. 1 for more details.

### Educational methods

We used two separate educational approaches in this teaching reform: one smart teaching method and one traditional teaching method. The Ministry of Education of the People’s Republic of China announced the “Education Informatization 2.0 Action Plan” in April 2018, outlining detailed requirements for implementing the “Action Plan for the Innovative Development of Intelligent Education,“ ushering in a new era in China’s educational system [6]. Professor Zhu Zhiting defines smart education as “the construction of a technology-integrated ecological learning environment and the cultivation of human-machine synergistic data intelligence, teaching intelligence, in accordance with the principles of precision, individuality, optimization, synergy, thinking, and creativity, teachers are able to implement highly effective teaching methods, and learners can obtain highly effective learning outcomes.“ We will cultivate students with strong character, strong action abilities, strong thinking abilities, and profound creative potential [7].

We established this smart class under the concept of using information technology tools. For each lesson, class information will be given in advance, and the teacher will log in to Rain Classroom using his/her cell phone to produce the two-dimensional barcode, and the students will use their cell phones to scan the two-dimensional barcode on the PPT to begin the lesson. Throughout the lesson, the teacher will distribute practice questions based on the knowledge points, including objective and subjective questions. The subjective questions are answered by randomly selected students using the Rain Classroom software. Students can view the PPT on their cell phones, and if they don’t understand something, they can operate the PPT on that page, such as clicking on “Have a question,“ and the teacher can view this information in the background data and do some explanations and answer questions as needed. All interactions with students, as well as students’ mastery of knowledge and customary grades, are accessible via background data. Students can post pop-ups during lectures, such as some viewpoints on the content and comments to teachers; some pop-ups are quite intriguing and humorous, increasing the attention and vividness of the classroom.

In general, the most important difference between smart education and traditional education is that the techniques of informatization are radically different, as is the experience provided to students. Students can watch the PPT and lecture video of the course after class for as long as they want, until the end of the semester. This is an excellent way for students to review what they have learned. In the traditional class, students review the major material from the textbook, notebook, or other materials.

### Survey subjects and statistical inspection

#### Survey subjects

The survey subjects are a total of 200 students in the pharmacy major (first semester of junior year) in the class of 2019. Satisfaction was measured by the Likert ten-point scale method, with a maximum score of 10 and a minimum score of 1 on the measurement scale. The lowest score indicates strong disagreement with the question’s description, while the highest score of 10 indicates strong agreement with the question’s description. The first and second questionnaires did not have the same content, and the second questionnaire focused on measuring effectiveness and satisfaction using the smart class method.

#### Scales and statistical tests

Firstly, we used descriptive statistics to examine the outcomes of some questions, and averages and standard deviations were used to express the results. Secondly, descriptive statistics were conducted, and statistical tests were run on the identical item “teacher’s teaching satisfaction” in both questionnaires. Thirdly, total satisfaction was divided into two secondary indicators: total teacher satisfaction and total class satisfaction, and four to five questions were assigned to each indicator, with a maximum of 10 points and a minimum of 1 point assigned to each issue of agreement. The total satisfaction data from the four groups of traditional classes and smart classes were tested for normality using IBM SPSS 23.0 software, P > 0.05 or P < 0.05, and combined with histograms to determine whether they were normally distributed. The Shapiro-Wilk test was used for total satisfaction data, and results showed P < 0.001 for four groups of data. None of the four total satisfaction data sets are normally distributed.So Mann-Whitney U test was taken.

## Results

### Student profile survey

#### Basic information of the interviewees

The respondents were 200 students from Zhengzhou University who took the Pharmacy Management course in the first semester of their junior year of undergraduate studies, with majors in undergraduate pharmacy and Pharmaceuticals. The first questionnaire survey collected 193 samples, accounting for 97.0% of the total proportion, and the second survey collected 199 samples, accounting for 99.5% of the total proportion. The number of male students among the respondents was 57, accounting for 29.5%, and the number of female students was 136, accounting for 70.5%, with a higher percentage of female students. Of the age factor, 87.6% were less than or equal to 21 years old. 12.4% of students were between 21 and 23 years old. See Table 1 for more details.

**Table 1:** Basic information about the respondents(the first survey)

		Numbers	Percentage
gender	male	57	29.5%
	famale	136	70.5%
	total	193	100%
age	18-21years old	169	87.6%
	21-23years old	24	12.4%
	Total	193	100%

**Table 2:** Respondents’ listening completeness and attention span(the first survey)

Content	Numbers	Percentage
A. Concentrate in class, pay attention, and completely listen to 90% or more of the class	66	34.2%
B. Your attention will be diverted to other places, listening completion rate is70%-90%	110	57.0%
C. Unable to pay full attention in class, listening completion rate is 50-69%	14	7.3%
D .listening completion rate is below 50%	3	1.5%
Total	193	NA

NA: Not Applicable

#### Respondents’ attention in class and influencing factors

According to the results in Tables 2 and 34.2% of the students were fully attentive and able to concentrate in class, 57% of the students completed 50–70% of the total lessons and 8.5% of the students completed 70% lessons.

The teacher factor was the primary influence in the survey of factors influencing attention in lectures, with 92.75% of students choosing this option, and the second factor was course and examination requirements, with 88.08% of students choosing this option. Self-motivation was chosen as the third factor by 86.01% of students. Objective factors such as whether the course is required, course examination requirements, and process evaluation have a greater impact on students’ attention in class. 129 students, accounting for 66.84% of the total number of students surveyed, chose the influence factors of classroom environment and hardware environment, such as multimedia.

### Teacher teaching satisfaction evaluation comparison between traditional class and smart class

The two questionnaires had different subject focuses, with the second section of the first survey evaluating course content and textbook and the corresponding part of the second survey evaluating the smart classroom. We investigated the findings of the replies to the component of the questionnaire that was the same between the two surveys: the evaluation of teacher satisfaction.

**Table 3:** Results of teacher teaching satisfaction evaluation on traditional and smart classes

Items	Traditional Classes Mean ± Standard deviation(n = 193)	Smart Classes Mean ± Standard deviation(n = 199)	Mann-Whitney U test	
			*Z*	*P*
This teacher has the skills of properly planning and teaching classes.	9.80$\pm$0.495	9.87$\pm$0.506	2.324	0.020*
key points and Difficult points are highlighted and the teacher is clear and careful.	9.74$\pm$0.667	9.82$\pm$0.647	2.031	0.042*
Teachers are knowledgeable and able to integrate theory into practice.	9.79$\pm$0.539	9.85$\pm$0.506	1.493	0.135
The content of the lecture can be combined with the licensed pharmacist examination.	9.82$\pm$0.500	9.82$\pm$0.545	0.253	0.800
Capability to educate students on the most recent research findings and content adjustments.	9.69$\pm$0.739	9.82$\pm$0.584	1.972	0.049*
Putonghua standard, fluent language, expressive, enjoyable lessons.	9.78$\pm$0.584	9.83$\pm$0.490	0.906	0.365

*P < 0.05

Table 3 shows that, with the exception of the identical score for the item “the content of the lecture can be combined with the licensed pharmacist examination,“ the scores of other five results of the smart classes were higher than those of the traditional classes. The Mann Whitney U Test was applied, and the findings are reported in Table 3. We received three positive outcomes, and each with a p-value less than 0.05.This indicates smart class teaching methods are more effective than traditional teaching methods in three aspects.

### Traditional class versus smart class satisfaction

First, for the four groups of satisfaction data variables, the normality test was performed in both surveys, and the test results were all P < 0.001. After combining the data histograms, it was determined that all four groups of data were skewed, so the Mann-Whitney U test was used to compare traditional and smart classroom satisfaction. The median of the four data sets, as well as 25% and 75% of the data, were assigned a score of 10. The overall satisfaction data was described using the mean standard deviation. In terms of overall teacher satisfaction, the statistical tests generated 1.331 for Z value and 0.183 for p-value, and 1.663 for Z value and 0.096 for p-value in terms of overall classroom satisfaction.

**Table 4:** Statistical test results of teacher satisfaction and class satisfaction

Types of satisfaction	Groups	Mean ± Standard deviation	Mann-Whitney U test	
			*Z*	*P*
Teacher satisfaction	traditional class(n = 193)	9.82 ± 0.471	1.331	0.183
	smart class(n = 199)	9.85 ± 0.566		
Class satisfaction	traditional class(n = 193)	9.68 ± 0.636	1.663	0.096
	smart class(n = 199)	9.75 ± 0.649		

Table 4 shows that both teacher satisfaction and class satisfaction scores improved after using the Smart Class, with an average increase of 0.03 points in teacher satisfaction and 0.07 points in class satisfaction compared to the traditional class. However, there is no significant difference in the average satisfaction scores between traditional class and smart class with the p-value is more than 0.05.

### Results of qualitative survey

The questionnaire’s subjective question is about the course’s recommendations and opinions. A total of 193 comments were received on the last question, including 79 “no comments or suggestions”. 114 comments are available for analysis and evaluation. We classified and summarized the 114 comments and the frequency numbers greater than or equal to 3 are shown in Table 5.

**Table 5:** Results of the qualitative analysis

Main content	Frequency
the teacher is very nice, responsible and serious, Pretty good, Especially good! Super! Class! Awesome! The teacher is so great!	37
Suggest replacing the classroom in the north core teaching building; the south core teaching area PPT cannot be seen clearly.	32
It is more convenient to view PPTs, replay courses, and access associated information in the rain classroom. More use of the rain classroom!	7
Thanks!Thank you for your efforts and hard work, I really love you!	7
The content of the book and the teacher do not always match the lecture; some content is not found in the book.	7
Very satisfied!	5
The PPT is not clear; the focus is not too prominent.	5
More interaction between teacher and student is recommended.	3
Teaching more with the latest cases and exercises.	3

## Discussion

### Satisfaction scores after using smart classroom instruction improved, but no statistical difference

After using Smart Class, both teacher and course satisfaction scores improved, and there were more comments from students’ subjective suggestions that they preferred Smart Class. No difference in statistical tests could be due to the use of 200 people in teaching arrangements to teach in large classes, and teaching methods such as flipping classrooms and group discussions are limited, which limits the effect of smart classrooms to some extent. Secondly, the multimedia classroom screen facilities in the south core teaching area are aging, the display is unclear, and the video and animation playback effect is affected by the slow internet speed. Lin Q [8] et al. article showed that there was no statistically significant difference in the final objective question scores and questionnaire results between the two groups of students in the smart class and traditional class groups (p = 0.874). However, data from student questionnaires and teacher interviews showed that students preferred to combine the smart class teaching module with the traditional class teaching module.

### Factors influenced attention in class

The survey results show that 57%of students will be distracted for part of the class and the attention may be transferred to other places, and the completeness of listening to the lesson ranges from 70 to 90%. 8.8% of students are unable to fully concentrate in class, and the completeness of the lesson ranges from 50 to 69% or less. In recent years, many college students are unable to concentrate because of factors such as mobile phones and postgraduate entrance examinations. As a result, it is critical to change teaching methods, cultivate teaching content deeply, stimulate students’ learning interest, and improve students’ attention. According to the survey results, the processing evaluation should be included in the final performance evaluation, which can motivate students to improve their attention, actively participate in the hall interaction, and thereby improve learning attention and teaching effects. All at the same, the rapid development of information technology has provided teachers with new challenges and learning opportunities. Teachers’ teaching abilities must also be continued to reform and optimize in order to match up to the current educational background [9].

### Teachers’ evaluation of smart class

High-quality Internet resources can be fully utilized with the use of Rain Classroom, and Internet + higher education resources are inserted in the chapters on pharmaceutical intellectual property management and pharmaceutical advertising management to broaden students’ horizons. At the same time, by borrowing information technology tools and means, teacher-student interaction becomes more convenient, and interaction can be increased in Rain Classroom through random roll call. With the addition of tasks linked to the knowledge points of the National Licensed Pharmacist Examination from the previous three years, the instructor can check the students’ mastery of the knowledge points for the first time and explain any deficiencies in detail. Students who provide excellent answers gain confidence in their upcoming pharmacist exam and improve their comprehension and memory of the knowledge points. The survey’s qualitative evaluation results confirm the quantitative evaluation results of greater satisfaction. Jin NB et al. [10] selected ten variables to evaluate the experience of smart classroom services: enjoyment, perceived interaction, subjective norms, privacy concerns, and computer anxiety. The findings revealed that, despite concerns about personal privacy and relatively low ratings in enjoyment and subjective norms, the smart class remains a viable tool.

## Conclusion

Teachers’ satisfaction and class satisfaction scores have improved after using smart class. When many indicators before and after intervention (i.e., traditional classrooms and smart classrooms) are compared, smart classroom scores are higher. However, in terms of overall satisfaction, there is no statistical difference between the average score of traditional class and smart class satisfaction before and after intervention. From the results of qualitative evaluation, the smart class is still popular with students. However, affected by the design of the curriculum, the study mainly adopt the Licket’s ten -point scale method, it is still a more subjective evaluation method. Objective evaluation instruments, such as performance analysis, are lacking, a variety of evaluation instruments and techniques will be used in further research.

## Data Availability

We are concerned that the disclosure of raw data may reveal the privacy of respondents, so we choose to make the following statement: The datasets used and/or analyzed during the current study are available from the corresponding author on reasonable request.

## Declarations

### Statement

Our survey protocol has been approved by the Ethics Committee of the College of Pharmaceutical Sciences, Zhengzhou University.
